# Splicing factor ratio as an index of epithelial-mesenchymal transition and tumor aggressiveness in breast cancer

**DOI:** 10.18632/oncotarget.13682

**Published:** 2016-11-29

**Authors:** Pietro Fici, Giulia Gallerani, Anne-Pierre Morel, Laura Mercatali, Toni Ibrahim, Emanuela Scarpi, Dino Amadori, Alain Puisieux, Michel Rigaud, Francesco Fabbri

**Affiliations:** ^1^ Biosciences Laboratory, Istituto Scientifico Romagnolo per lo Studio e la Cura dei Tumori (IRST) IRCCS, Meldola (FC), Italy; ^2^ Inserm UMR-S1052, Centre de Recherche en Cancérologie de Lyon, Lyon, France; ^3^ CNRS UMR5286, Centre de Recherche en Cancérologie de Lyon, Lyon, France; ^4^ Centre Léon Bérard, Lyon, France; ^5^ UNIV UMR1052, Lyon, France; ^6^ Université de Lyon, Lyon, France; ^7^ Osteoncology and Rare Tumors Center, Istituto Scientifico Romagnolo per lo Studio e la Cura dei Tumori (IRST) IRCCS, Meldola, Italy; ^8^ Unit of Biostatistics and Clinical Trials, Istituto Scientifico Romagnolo per lo Studio e la Cura dei Tumori (IRST) IRCCS, Meldola, FC, Italy; ^9^ Department of Medical Oncology, Istituto Scientifico Romagnolo per lo Studio e la Cura dei Tumori (IRST) IRCCS, Meldola, FC, Italy; ^10^ Institut Universitaire de France, Paris, France

**Keywords:** EMT, early breast cancer, tumor aggressiveness, alternative splicing, EMT ratio

## Abstract

Epithelial-to-mesenchymal transition (EMT) has been shown to be associated with tumor progression and metastasis. During this process in breast cancer, a crucial role is played by alternative splicing systems. To identify a new early prognostic marker of metastasis, we evaluated EMT-related gene expression in breast cell lines, and in primary tumor tissue from 31 patients with early breast cancer, focusing our attention on EMT-related splicing factors ESRP1, ESRP2 and RBFOX2. Results showed that the expression patterns of these genes were indicative of the onset of EMT in *in-vitro* models, but not in tissue samples. However, the ratio between ESRP1 or ESRP2 and RBFOX2 significantly decreased during EMT and positively correlated with the EMT-specific phenotype in cell models, representing a promising prognostic markers. Low ESRP1/RBFOX2 ratio value was associated with a higher risk of metastasis (*p* < 0.005) in early breast cancer patients, regardless other clinical features. A cut-off of ratio of 1.067 was determined by ROC curve analysis (AUC 0.8375; 95% CI 0.6963–0.9787). Our study show evidence that a decrease in this ratio correlates with cancer progression. The results provide a rationale for using ESRP1/RBFOX2 ratio as a new prognostic biomarker for the early prediction of metastatic potential in breast cancer.

## INTRODUCTION

Breast cancer is the most frequently diagnosed malignancy and the second cause of cancer-related mortality in the female population [1, 2]. Despite advances made in the management of this disease over the past few decades, up to 20–25% of patients with early breast cancer relapse within 5 years of diagnosis [1, 3]. Given that metastasis is the leading cause of cancer-related death, it is essential to understand the biological process characterizing this phenomenon in an attempt to detect and counteract a potential relapse, especially in the initial phases of disease progression.

Metastasis, defined as the spreading of cancer cells from the primary tumor to the blood circulation or lymphatic system and their migration to distant organs, is a complex and multi-step process that can occur at any stage of the disease, including earlier stages [4]. In order to succeed in such an arduous endeavour, cancer cells have to substantially modify a number of their essential features [5].

Epithelial-mesenchymal transition (EMT), is a reversible normal morphogenetic process capable of converting polarized epithelial cells into mesenchymal cells [6], involved in embryogenesis, wound healing processes and chronic diseases such as fibrosis. In cancer, EMT may be involved in disease progression, by driving cells towards a more aggressive phenotype [6–8]. EMT induces the loss of cell junctions [9] and of apical-basal polarity organization [10], enabling cell motility. These phenomena allow tumor cells to invade stromal tissue, enter the peripheral blood flow as circulating tumor cells (CTCs) where they resist physical and biochemical stress, and ultimately generate distant metastases by the opposite process known as mesenchymal-epithelial transition [7, 11–13]. It is also known that EMT confers stemness features to cancer cells such as growth arrest and resistance to senescence, apoptosis and chemotherapy [14–17]. EMT-associated reprogramming is closely controlled by a complex regulatory network at both transcriptional and translational levels comprising four main interconnected systems [18]. These systems include transcriptional factors (TFs), non-coding RNAs, alternative splicing factors and other post-translational controls. Among these, the alternative pre-RNA splicing system has recently aroused interest after experimental results suggested that splicing control may play an important role in the onset and progression of cancer [19–22].

EMT-associated alternative splicing events in cancer have recently been described in the literature, indicating that transcriptome-wide remodelling correlates with more aggressive features [23, 24]. In epithelial cells the master splicing regulators are epithelial-specific splicing factor 1 (ESRP1) and 2 (ESRP2) [25, 26]. In contrast, the RNA binding protein fox-1 homologous (C. elegans) 2, (RBFOX2) has been shown to be a driving factor of mesenchymal-related splicing in normal and cancer tissue [27]. With regard to the link between EMT and tumor progression, aggressiveness and poor clinical outcome, recent reports have shown that EMT-linked alternative splicing patterns may facilitate the identification of aggressive tumor variants, especially in breast, lung and colon cancer [23, 24]. However, research is still at a preclinical stage and a specific pattern of splicing factor expression has not yet been clearly linked to prognosis.

In this work we investigated the ratio between epithelial- and mesenchymal-specific splicing factors as a potential EMT-based early marker of tumor aggressiveness in early stage breast cancer. The study was performed on two EMT-induced cell lines, MCF10A and HMEi-SNAIL, and in primary early breast cancer tumor tissues. Results on *in vitro* EMT models indicated that the two ratios between the phenotype-specific splicing factors, ESRP1/RBFOX2 and ESRP2/RBFOX2 significantly decreased during EMT, correlating positively with the EMT-specific phenotype. Moreover, their investigation in fresh primary tumor tissue from patients with early breast cancer revealed that a low ESRP1/RBFOX2 ratio value was significantly associated with a high risk of metastasis in early breast cancer.

## RESULTS

### EMT features in cell lines

**Figure 1:**
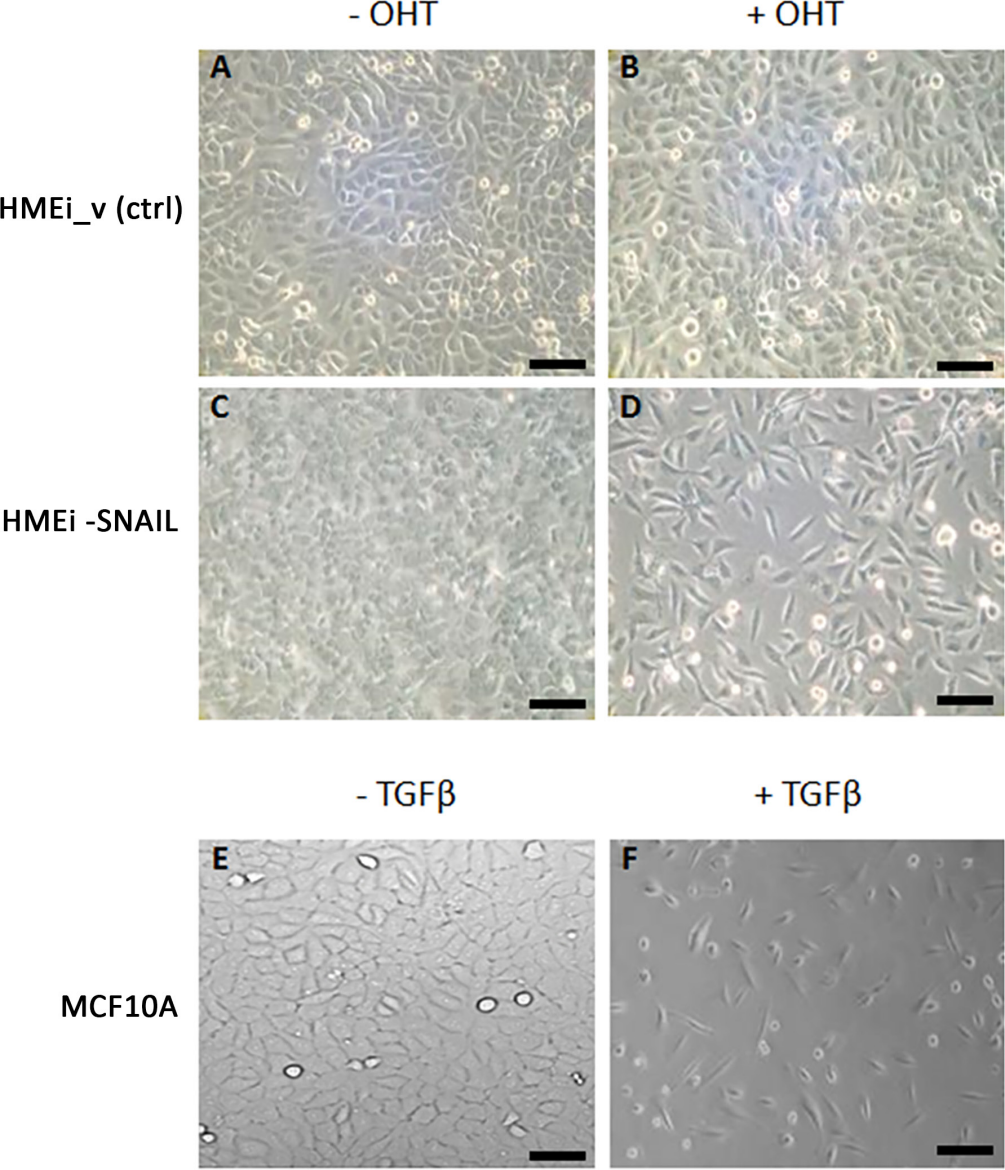
Phase-contrast images of MCF10A, HMEC immortalized with empty vector (HMEi_v) and HMEi-SNAIL cell cultures (**A**) HMEi_v cells without 4-OHT; (**B**) HMEi_v cells treated with 4-OHT; (**C**) HMEi-SNAIL cells without 4-OHT (control); (**D**) HMEi-SNAIL cells at the end of 4-OHT treatment (13^th^ day); (**E**) MCF10A cells without TGFβ (control); (**F**) MCF10A cells at the end of TGFβ treatment (13^th^ day). Magnification 10× for all images (scale bar: 50 μm).

EMT was induced in HMEi-SNAIL and MCF10A cell lines by the overexpression of the inducible form of EMT-TF SNAI1 (hSNAIL-ER) and by TGFb treatment, respectively. Untreated cells showed tightly packed clusters with a typical epithelial phenotype, *e.g*. tight cell-to-cell adhesion. These cells maintained a standard doubling time of 48 hours. After EMT induction, morphological EMT-related changes were observed in both cell lines. The peculiar epithelial tissue-like structure disappeared and the distinctive cobblestone-like organization was lost in favour of a spindle-like phenotype and a characteristic fibroblast-like morphology. An increase in doubling time of up to 72 hours was also observed. In HMEi-SNAIL cells, mesenchymal features appeared after 5–6 days’ induction, whereas morphological changes in MCF10A cells emerged no more than 4 days after the start of treatment. After 13 days, both cell lines showed a complete mesenchymal-like phenotype (Figure 1). Immortalized HMECs with empty vector (HMEi_v) did not acquire EMT-like features after treatment with 4-OHT, maintaining control sample characteristics (Figure 1A and 1B).

### EMT gene expression in cell models

#### Epithelial and mesenchymal genes

**Figure 2:**
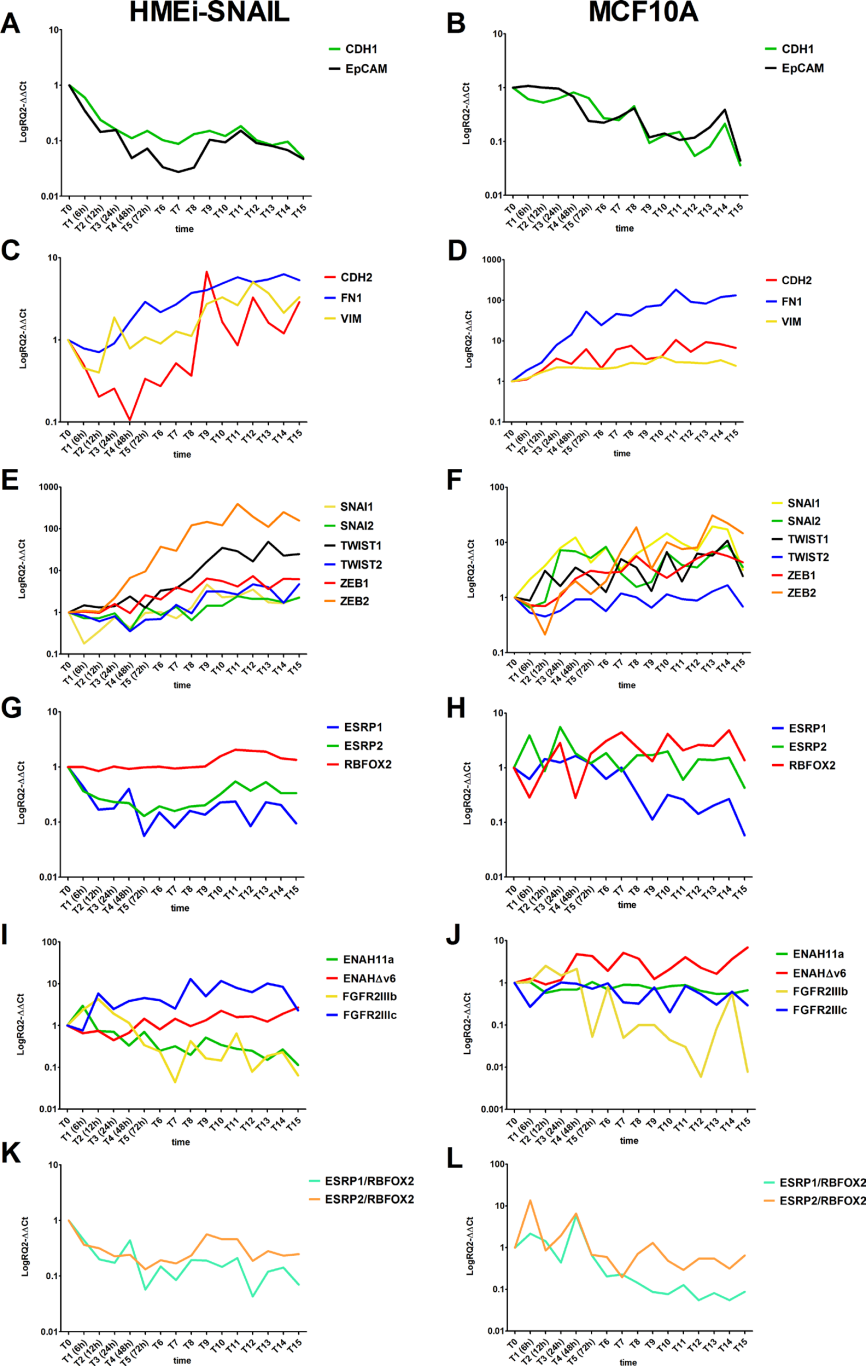
Kinetics of EMT-related gene expression in treated HMEi-SNAIL (left) and MCF10A (right) in which EMT was induced Epithelial gene expression in HMEi-SNAIL (**A**) and MCF10A (**B**); Mesenchymal gene expression in HMEi-SNAIL (**C**) and MCF10A (**D**); EMT-TF expression in HMEi-SNAIL (**E**) and MCF10A (**F**); EMT-related splicing factor expression in HMEi-SNAIL (**G**) and MCF10A (**H**); EMT-related splicing factor expression ratio values in HMEi-SNAIL (**I**) and MCF10A (**J**); Splicing variant products in HMEi-SNAIL (**K**) and MCF10A (**L**). Cell samples were collected and analyzed every 6 hours during the first 24 hours (from T0 to T3). From 24 hours to the 13th day of treatment (T15), cells were detached and tested every 24 hours.

In HMEi-SNAIL cells, E-cadherin (CDH-1) was downregulated about 6-fold after only 6–24 hours (T2-T4), decreasing up to 20-fold at later time-points (Figure 2A). In MCF10A, CDH-1 expression also decreased after 6 h but showed a heavy downregulation only after 4 days (T6), further decreasing up to 23-fold after 13 days (T15) (Figure 2B). EpCAM was downregulated already during the early phases of the transition, *i.e.* after 6 hours (T2) and 24 hours (T4), in HMEi-SNAIL and MCF10A respectively, decreasing by ~20-fold after seven days (T9) in both cell lines (Figure 2A and 2B). Thus, epithelial genes showed a substantial downregulation compared with mesenchymal genes, which were upregulated. Vimentin (VIM) expression was clearly enhanced after only 24 hours in both models, increasing up to 3.5-fold after seven days (T9) in HMEi-SNAIL cells and no more than 4-fold in MCF10A (Figure 2C and 2D, respectively). N-cadherin (CDH-2) and fibronectin-1 (FN1) showed a similar trend. In HMEi-SNAIL cells, CDH-2 expression decreased up to day 2 (T4) but then increased up to 8-fold by day 7 (T9), showing a variable trend with an increased expression after each treatment, *i.e.* every 48 hours (Figure 2C). In MCF10A, CDH-2 was upregulated 3-4-fold after 24 hours, further increasing by up to ~11-fold at the last few experimental times (Figure 2D). FN1 was upregulated right from the early phases of the transition (48 hours after the first treatment) in both cell lines. In HMEi-SNAIL cells, FN1 expression did not increase more than 6-fold, (Figure 2C), whereas in MCF10A it showed an increase of up to 100-fold (Figure 2D).

### EMT-related TFs

The expression of almost all the EMT-TFs was upregulated in both treated lines. In HMEi-SNAIL cells, an increase in TWIST2 expression was only visible from the 5^th^ day (T7) of treatment onwards, while ZEB2 showed a 4-fold upregulation already after 24 hours (T3), increasing up to 200-fold after 13 days (T15). TWIST1 and ZEB1 expression levels increased progressively during the transition process, whereas SNAI2 expression remained unvaried for the first 7 days (up to T9), subsequently increasing up to ~2.5-fold. Endogenous SNAI1 showed low expression for the first 5 days of treatment and increased slightly from day 6 (T8) onwards (Figure 2E). In MCF10A, SNAI1 showed a more than 12-fold higher expression 24 hours after the start of EMT induction than that of untreated samples, with a variable expression that spiked after each TGF-b treatment (Figure 2F). TWIST1, SNAI2 and ZEB1 increased after 12 hours (T2), 24 hours (T3) and 48 hours (T4), respectively, but displayed a more gradual upregulation during EMT, increasing no more than 10-fold with respect to controls (Figure 2F). ZEB2 expression changed dramatically after 24 hours (T3), increasing over ~14-fold (Figure 2F). TWIST2, on the other hand, did not show any upregulation (Figure 2F).

### Splicing factors

ESRP1, ESRP2 and RBFOX2 showed variations in expression during EMT. In HMEi-SNAIL cells, ESRP1 and 2 were downregulated from the early stages of treatment (from 6 to 12 hours), maintaining low expression levels (6- to 12-fold below baseline) throughout the EMT process (Figure 2G). In MCF10A, only ESRP1 was substantially downregulated (13-fold) after only 24 hours (T3) and remained so for all of the experimental time-points. ESRP2 simply showed a decline in expression after each TGF-β treatment (Figure 2H). RBFOX2 behaved differently in both cell models (Figure 2G and 2H). In HMEi-SNAIL cells, this marker showed a late and gradual upregulation in expression not exceeding 3- to 5-fold after 7 days’ treatment (Figure 2G). In MCF10A, RBFOX2 was already upregulated after 24 hours (T3), with expression levels varying during subsequent time points but never increasing more than 5-fold (Figure 2H).

### Phenotype-related splicing variants

Epithelial-specific splicing variants, ENAH11a and FGFR2IIIb, were gradually downregulated in both EMT models, decreasing by up to 8.7-fold after 13 days (T15) and up to 22-fold after 5 days (T7), respectively, in HMEi-SNAIL cells (Figure 2I). In TGF-β-treated MCF10A cells, ENAH11a was 3.7-fold downregulated from 12 hours (T2) onwards, while FGFR2IIIb decreased up to 120-fold at the end of the EMT induction period (Figure 2J). In contrast, the mesenchymal variants were upregulated. In HMEi-SNAIL cells, ENAHΔv6 expression was slightly upregulated after 72 hours (T5), reaching 2.7-fold more at the end of treatment, while FGFR2IIIc showed a 5.8-fold upregulation after 12 hours, increasing up to 11-fold after 6 days (T8) (Figure 2I). In MCF10A cells, ENAHΔv6 expression showed a 2-fold increase after only 6 hours (T1) but then fluctuated throughout the rest of the EMT-induction period, whereas FGFR2IIIc did not show any clear upregulation (Figure 2J).

### Splicing factor ratios

We analyzed the ratio between ESRP1 or ESRP2 and RBFOX2 mRNA levels. With respect to untreated controls, the ratio between either ESRP and RBFOX2 showed a time-related decrease of up to about 5-fold (Figure 2K and 2L) in both EMT models. This value was reached within 24 hours of the first treatment and was maintained at all the subsequent experimental time-points.

### EMT gene expression in early breast cancer tissues

**Figure 3:**
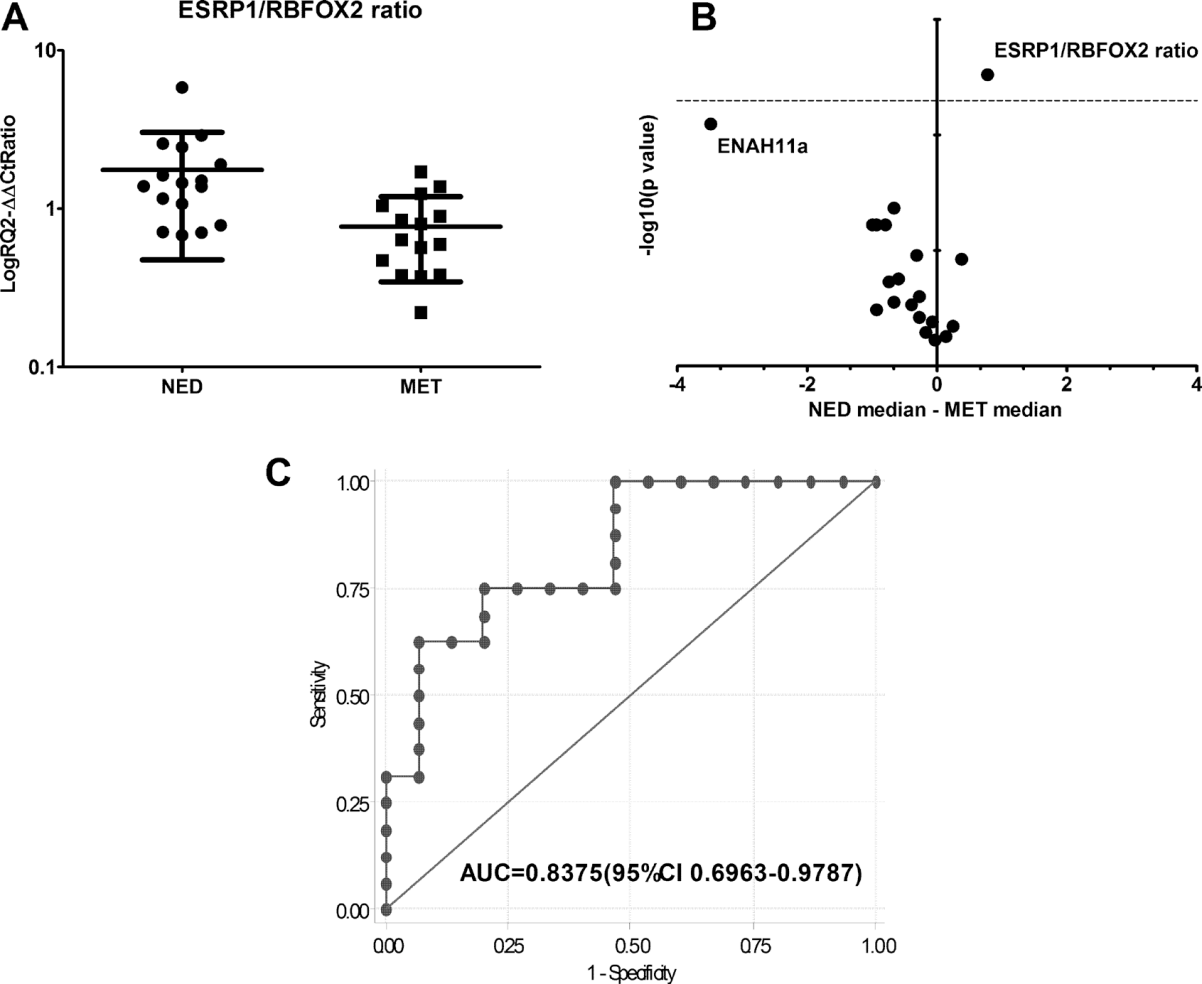
(**A**) ESRP1/RBFOX2 gene expression ratio values in NED and MET samples (*P ≤ 0.005). (**B**) Volcano plot representing the differences in median expression levels between NED and MET samples plotted against their statistical significance for all assays. The ESRP1/RBFOX2 ratio is the only assay with a statistical power ratio that lies above the horizontal threshold line (dashed line: *P* = 0.005). *T-test P* values of the comparison samples are shown in Table 1. (**C**) ROC curve of ESRP1/RBFOX2 ratio between NED and MET samples and (AUC = area under curve).

In order to investigate whether the EMT events observed in the *in vitro* models correlated with aggressiveness in early breast cancer, we analyzed fresh tumor tissue samples from 31 patients with primary breast cancer collected and stored at time of diagnosis and before the treatment (from 1997 to 2000). Patient samples were divided into two subgroups on the base of a minimum follow-up of 10 years: patients with metastatic disease (MET) and subjects with no evidence of disease (NED). No significant differences in the expression level of single EMT-related markers were observed between the two groups (Table 1). However, the EMT-TF TWIST1 and ZEB2 showed a weak upregulation in MET samples with respect to NED samples. Furthermore, VIM and FN1 showed higher, albeit not significantly, median expression levels in MET than in NED samples (Table 1). The analysis of single splicing factors ESRP1, ESRP2 and RBFOX2 did not reveal any differences between the 2 groups (Table 1). However, the ratio between the expression values of the alternative splicing factor ESRP1 and RBFOX2 were significantly different between NED and MET samples (*P* < 0.005) (Table 1 and Figure 3A). This ratio was statistically significant after FDR correction. We also analyzed the phenotype-specific splicing products of ENAH and FGFR2 but did not observe significant differences between the two subgroups for either of these genes Table 1. Furthermore, we performed a volcano plot analysis, comparing the differences in median expression values between subgroups (x-axis) with the corresponding level of statistical significance (-log10 *p-value*; y-axis) to show the statistical power of the assays evaluated here (Figure 3B). The ESRP1/RBFOX2 ratio was the only test that was statistically significant, inferring a value clearly over the point-of-interest (dashed line) (Figure 3B). ROC curve analysis for ESRP1/RBFOX2 ratio was performed (Figure 3C). The ratio ESRP1/RBFOX2 showed fairly high specificity in discriminating between breast cancer tissue from NED patients and that from MET patients, with an area under ROC curve (AUC) of 0.8375 (95% CI 0.6963–0.9787), (Figure 3C and Table 1). ROC curve analyses identified the best cut-off, showing that breast tumor tissue samples with a ratio ≥ 1.069 were NED (low risk of metastasis), while those with a ratio < 1.069 were MET (high risk). The accuracy of prediction was 78% (95% CI 63–92), with a sensitivity of 75% (95% CI 54–96) and a specificity of 80% (95% CI 60–100).

**Table 1:** Median gene expression values of EMT-related genes and median ratio value ESRP1/RBFOX2 and ESRP2/RBFOX2 in early breast cancer tissue

	NED (*n* = 16)	MET (*n* = 15)	
Assay	Median value (range)	Median value (range)	*Ps*
**SNAI1**	0.89 (0.32–13.92)	0.64 (0.19–6.36)	0.653
**SNAI2**	0.80 (0.19–3.83)	1.07 (0.30–6.49)	0.545
**TWIST1**	1.18 (0.17–2.43)	1.21 (0.20–3.76)	0.860
**TWIST2**	0.78 (0.13–10.10)	1.71 (0.41–4.01)	0.060
**ZEB1**	0.83 (0.09–6.15)	1.57 (0.33–19.19)	0.269
**ZEB2**	0.66 (0.14–2.42)	1.45 (0.41–6.56)	0.060
**CDH1**	0.81 (0.14–14.15)	1.08 (0.35–3.35)	0.360
**EPCAM**	0.79 (0.19–5.58)	0.96 (0.40–7.59)	0.739
**CDH2**	1.03 (0.28–7.54)	1.10 (0.08–25.88)	0.597
**VIM**	0.79 (0.18–4.23)	1.18 (0.16–4.95)	0.424
**FN1**	0.75 (0.20–6.94)	1.41 (0.11–6.05)	0.402
**ESRP1**	1.14 (0.42–6.72)	0.76 (0.36–2.09)	0.171
**ESRP2**	0.92 (0.12–17.84)	1.23 (0.45–3.47)	0.159
**RBFOX2**	0.74 (0.26–4.63)	1.40 (0.66–2.61)	0.043
**FGFR2IIIb**	1.37 (0.06–3.03)	2.30 (0.02–10.98)	0.470
**FGFR2IIIc**	0.97 (0.13–2.79)	1.56 (0.13–13.80)	0.253
**ENAH11a**	0.43 (0.003–8.86)	3.91 (0.28–13.85)	0.008
**ENAHdV6**	0.43 (0.01–9.37)	1.42 (0.72–28.16)	0.060
**Ratio ESRP1_RBFOX2**	1.42 (0.68–5.82)	0.64 (0.22–1.71)	0.003*
**Ratio ESRP2_RBFOX2**	1.02 (0.10–4.09)	0.88 (0.36–2.69)	0.799

* *P* ≤ 0.005.

### Immunohistochemistry analysis

**Figure 4:**
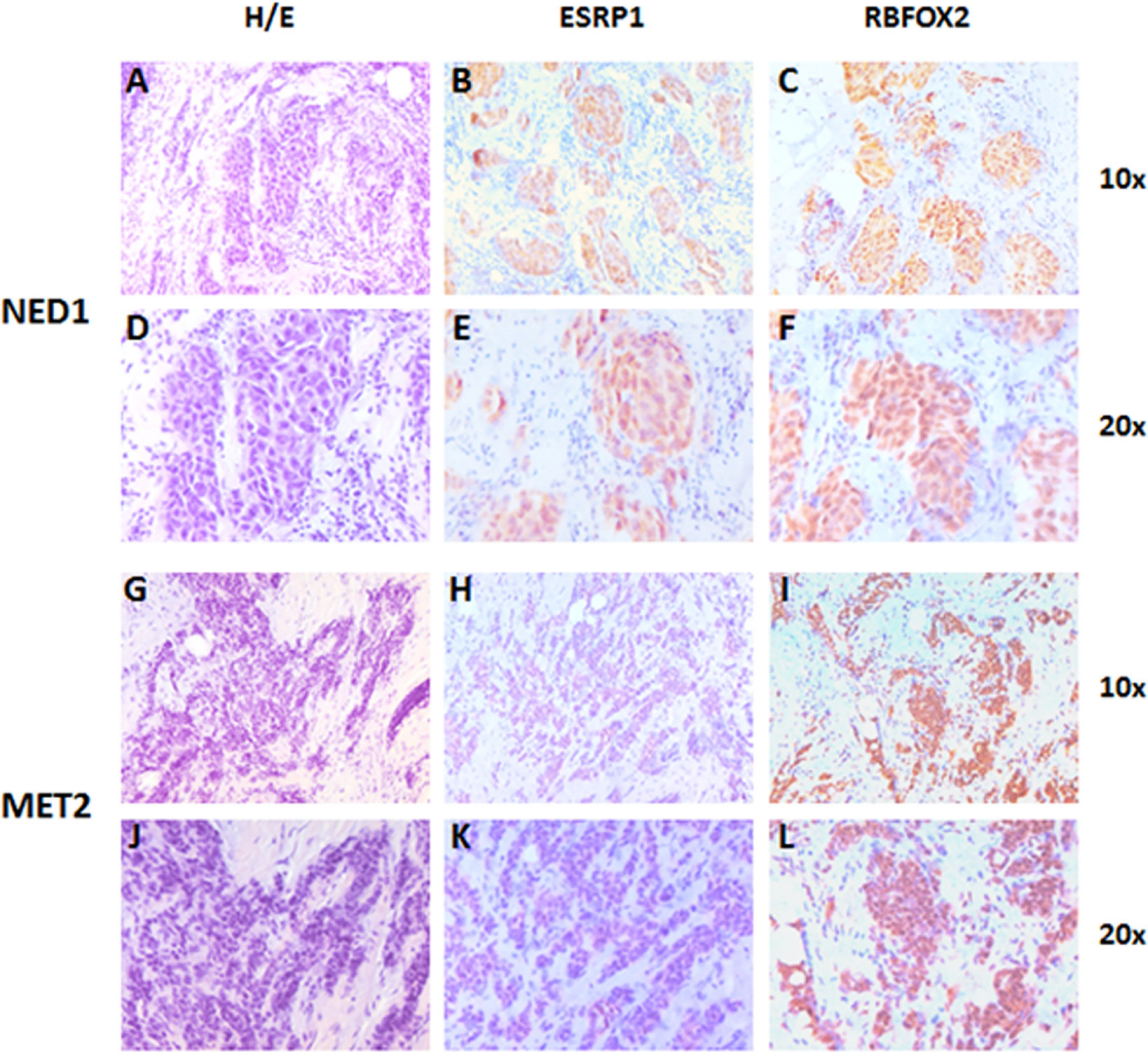
Representative images of ESRP1 and RBFOX2 expression in early breast cancer tissue of NED 1 (T2N0M0 at time of diagnosis) and MET 2 samples (T2N0M0 at time of diagnosis) Two ductal infiltrating tumor samples of grade two. (**A** and **D**) Hematoxylin and eosin staining of NED 1 sample; (**B** and **E**) ESRP1 immunostaining of NED 1 sample (grade 3); (**C** and **F**) RBFOX2 immunostaining of NED 1 sample (grade 3); (**G** and **J**) Hematoxylin and eosin staining of MET 2 sample; (**H** and **K**) ESRP1 Immunostaining of MET 2 sample (grade 1-); (**I** and **L**) RBFOX2 immunostaining of MET 2 sample (grade 3+). Magnification 10× and 20×.

To evaluate whether changes observed at the RNA level of ESRP1 and RBFOX2 were translated at the protein level, we performed an immunohistochemistry assay on 4 FFPE tissue samples from patients with stage 1, grade 2 infiltrating ductal breast cancer. The analysis confirmed the presence of both ESRP1 and RBFOX2 protein expression in all tumor cell compartments, especially the nucleus. ESRP1 was poorly expressed in MET (semi-quantitative level of expression grade 1/1+ and 2 in MET 1 and 2, respectively) with respect to NED (semi-quantitative level of expression grade 3 in both samples). RBFOX2 expression did not vary substantially among tumor areas, but it was slightly higher in MET 1 and 2 (grade 3 and 3+, respectively) than in NED 1 and 2 samples (grade 3 and 3, respectively). Images of two of the four FFPE samples analyzed are shown in Figure 4.

## DISCUSSION

There is increasing evidence pointing to the role of EMT in cancer progression, onset of metastasis and resistance to treatment [8]. The determination of an EMT-related gene expression pattern in the initial stages of tumor development could help to delineate a signature of “tumor aggressiveness” thereby leading to identify a highly hostile tumor, even at the earlier stages of disease. To address this issue we investigated into gene expression alterations using 2 *in vitro* models of EMT and primary tumor tissue from early breast cancers patients, focusing our attention on phenotype-specific splicing events.

We first studied EMT events in EMT-inducible HMEi-SNAIL and TGF-β-treated MCF10A, following two ways of transitions with the activation of different but some shared representative pathways [15, 17]. HMEi-SNAILs, immortalized human mammary epithelial cells, were modified to obtain a direct ER-driven inducible activation of SNAI1 by 4-OHT treatment, triggering EMT. Long-term treatment with recombinant TGF-β induced a well-defined mesenchymal phenotype in MCF10A, as previously reported [28]. These two models helped us to understand which factors are the most indicative of EMT temporal regulation among EMT-TFs, EMT-related genes and splicing factors.

In our study we observed well-known EMT features, including morphological and molecular changes, in both models. At the cellular level, these events initially lead to a growth delay, probably triggered by a profound reorganization of the cytoskeleton which is incompatible with a high proliferation rate [29]. Despite the similarities, a difference in the times of EMT occurrence was detected.

At the molecular level, gene expression analysis confirmed that the transition was ongoing in our cell models and started before morphological events took place, in agreement with other studies [18, 30]. Analyzing the early phases of the transition, we observed differences in the expression levels of EMT-markers between the models. We noticed that these differences occurred at different times during the transition process.

These cellular and molecular differences were probably linked to intrinsic peculiarities of the cell models. In HMEi-SNAIL cells, the activation of SNAI1 triggered a transition of cells towards a mesenchymal phenotype in a slower but more profound manner, influencing its direct targets of SNAI1 [31], and involving the emergence of stem-like properties, as already suggested by Mani et al. [17]. Conversely, MCF10A cells exhibit a basal-like phenotype with mesenchymal-like features and can be easily induced to undergo EMT [32]. TGF-β stimulation rapidly induces the upregulation of different EMT-TFs by the activation of SMAD proteins, stimulating SNAI1 and TWIST1 expression [31, 33, 34] which triggers the transition process right from the first treatment. Thus, both cell lines appeared to be valid EMT models for the study of early phases of EMT, and for the possibility to test novel EMT markers. Moreover, on the basis of these preclinical data, we hypothesized that EMT analysis of tumor tissue using conventional EMT markers could be compromised by the potential negative influence of the time of sample collection. EMT is a dynamic, reversible process that may occur in only a subset of cells or in specific regions of tumor tissue [35, 36]. Indeed, it is a very difficult process to detect, especially in cancer tissue samples that have not been microdissected. Taken together, these results highlight the potential usefulness of identifying an easily evaluable, stable EMT-based prognostic marker of tumor dissemination that can be monitored from the beginning of EMT.

In our models, ESRP1 and 2 were downregulated (especially ESRP1) in both cell lines even a few hours after the start of the induction, whereas RBFOX2 was weakly but stably upregulated, in agreement with precedent studies [23, 25]. ESRPs are the most important epithelial-specific genes capable of inducing a complete phenotypic cellular switch during EMT by their down-regulation [25, 37, 38]. ESRP1 and 2 expression are linked to low cell motility, and high level of ESRP1 in pancreatic cancer patients, correlating with a favourable prognosis [37, 39]. RBFOX2 plays a dual role in that it is both an important co-regulator of ESRP1 in epithelial phenotype cells and a mesenchymal-specific splicing factor during EMT and in more aggressive breast cancer subtype cell lines [23, 40]. In our study, their involvement was demonstrated through the expression analysis of splicing variants of their targets ENAH and FGFR2. These showed an upregulation of the mesenchymal variants ENAHdv6 [41] and FGFR2IIIc [42], and a downregulation of ENAH^11a^ [41] and FGFR2IIIb [42], confirming once again that the EMT process was ongoing at each investigated regulatory network level. In a recent work, Shapiro and colleagues [23] correlated specific alternative splicing patterns with the breast cancer phenotype and with known molecular features of the tissue analyzed. However, they did not investigate their potential correlation with prognosis.

Other studies have suggested that an “EMT score” could be based on a pattern or ratio of gene expression variations [32, 36, 43, 44]. A well combined approach was defined by Schliekelman et al. who proposed an EMT score based on the ratio between CDH1 protein, localized on the cell surface (CDH1_S), and total Vim protein expression (CDH1_S/Vim) in a lung cancer setting. The authors demostrated the possibility of distinguishing the epithelial state from the mesenchymal one or from a hybrid condition using a ratio between these two phenotype- specific genes [44].

On the basis of these assumptions, we tested two ratios between the epithelial and mesenchymal specific splicing factors, ESRP1/RBFOX2 and ESRP2/RBFOX2 in an early breast cancer setting. In *in vitro* experiments, both ratios showed a decreasing value from the initial stages of EMT that preceded the appearance of an EMT gene expression pattern, with a similar trend among the tested cell models. These data, in agreement with those in the literature [23, 24, 44], suggest that these two splicing factors, in regulating the splicing phenomenon of cancer cells, are capable of revealing the onset of the EMT process, also at an early stage, independently of the study models used in *in vitro* experiments. Thus, the ratio seems to predict the EMT process earlier than markers based on classic EMT targets.

Taken into account these preliminary results, we conducted a retrospective study in a cohort of 31 early breast cancer patients in which we analyzed the expression levels of the same EMT-related genes used in the *in vitro* study, with the aim to test the value of the ratio in a clinical setting and if it could correlate this with the prognosis. No one gene assay revealed a significant difference between MET and NED patients. ZEB2 gene showed a high, albeit not significant, expression level in patients with metastasis. Interestingly, the ratio between the alternative splicing factor ESRP1 and RBFOX2 highlighted a significant difference between NED and MET groups. This suggests that even a low fold-change value in the ESRP1/RBFOX2 ratio correlates with a high risk of metastasis, regardless of tumor grade, receptor status or any other clinical feature. These results enabled us to identify a ratio cut-off value with fairly good sensitivity (75%) and specificity (80%) that was capable of discriminating between a high (< 1.067) and low (> 1.067) risk of metastasis in breast cancer. Immunohistochemistry analysis confirmed that ESRP1 and RBFOX2 protein were expressed in all tumor tissue areas, in agreement with literature data [5, 45, 46], but with different intensity and distribution. In NED tissue, both proteins were highly and homogeneously expressed, whereas very low levels of ESRP1 and slightly higher levels of RBFOX2 were detected in MET tissue.

In conclusion, in agreement with previous results describing a correlation between EMT status and tumor aggressiveness, our findings confirmed that EMT can be linked to tumor progression. Although different studies have already investigated EMT-specific gene expression patterns including EMT-related splicing factors and alternative splicing events [24], few have analyzed transition phenomena in early stages of breast cancer progression [47, 48]. Testing the ESRP1/RBFOX2 ratio for the first time, we believe that we have identified a promising new prognostic biomarker for the timely prediction of the risk of metastasization, without the need of microdissection. It is also tempting to hypothesize that this ratio could be used in combination with CTC investigations to non-invasively define the nature of a tumor even when the primary disease is no longer present [49]. Although further studies and clinical validation steps in larger cohorts of patients are needed before passing from *bench to bedside*, our findings show that important information on the risk of metastasis can be drawn from EMT. Such information could be used to facilitate patient stratification and improve the effectiveness of the therapeutic intervention.

## MATERIALS AND METHODS

### Cell lines

Primary human mammary epithelial cells (HMECs) immortalized with hTERT [14], HMEi_v and HMEi-SNAILs cells (HMEC derivative cells), and MCF10A cell lines were used. The commercial cell lines MCF10A and HMEC cell line were purchased from the American Type Culture Collection (ATCC) in March 2009, and from Lonza (Lonza Group Ltd.) in June 2008, respectively. HMEi-SNAILs were generated by the Puisieux Laboratories, as described below.

HMECs, HMEi_v and HMEi-SNAIL were cultured in 1:1 Dulbecco's Modified Eagle's Medium (DMEM)/HAMF12 medium (Invitrogen) complemented with 10% FBS (Cambrex), 100 U/ml penicillin-streptomycin (Invitrogen), 2 mM L-glutamine (Invitrogen), 10 ng/ml human epidermal growth factor (EGF) (PromoCell), 0.5 mg/ml hydrocortisone (Sigma) and 10 mg/ml insulin (Actrapid). Subculture were maintained by puromycin (0.5 mg/ml). The commercial MCF10A cell line were maintained in culture as recommended by Soule and colleagues [50], in 1:1 DMEM/F12: 500 ml complemented with 5% Horse Serum (Invitrogen), 100 mg/ml EGF (Millipore), 1mg/ml Hydrocortizone (Sigma), 1mg/ml CholeraToxin (Sigma), 10mg/ml Insulin (Sigma), 100 U/mL Pen/Strep (EuroClone). All cell lines were maintained in a 37°C incubator with 5% CO2 and subcultured weekly. Cells in culture have been seeded a 1*10^6^ in a dishes of 10 cm^2^, with 10 ml of specific medium described. Original cell lines were authenticated by STR profiles and were used for experiments within 25 passages.

### Lentiviral and retroviral infections

HMEi-SNAILs and HMEi_v (control, with empty vector) were generated by transfecting Phoenix cells [51] with 15 μg of retroviral expression vectors (pBabe-hygro-hTERT, pBabe puro/hSNAIL-ER or pBabe puro empty vector) using calcium-phosphate precipitation. Forty-eight hours post-transfection, the supernatant was collected, filtered, supplemented with 5 μg/ml of polybrene (Sigma) and combined with 10^6^ target cell HMECs for 6 hours. Cells were infected twice and selected 48 hours after the second infection with hygromycin (10 μg/ml) and puromycin (0.5 mg/ml), as described by Morel et al. [15].

### EMT induction methods, morphological examinations and sample collection

To study the early stages of EMT and to define a “signature of aggressiveness”, we used two distinct *in vitro* breast cell line models of EMT induction, HMEi-SNAIL and MCF10A, in which we evaluated EMT development by analyzing morphological change and the expression of a panel of phenotype-specific and EMT-related genes. HMEi-SNAIL cells were treated with 40 nM of 4-hydroxy tamoxifen (4-OHT, ER ligand) every 48 hours for 16 days to study direct EMT induction through the expression and activation of SNAI1 (SNAIL), the principal EMT-TF [17, 46]. MCF10A cells were treated with 10 ng/ml of the recombinant cytokine TGFβ1 (Peprotech) every 48 hours for 16 days to mimic physiological induction of EMT. Morphological examination of both cell lines was performed by light microscopy Axiovert 200 (Zeiss) every 24 hours. Cell samples for molecular analysis were collected at established time-points, every 6 hours during the first day and every 24 hours thereafter throughout the 13-day EMT induction period. All analyses were performed at each time-point.

### Patient enrolment

A retrospective study was performed on 31 female early breast cancer patients selected from a case series consecutively enrolled from 1997 to 2000. Patients aged ≥ 18 years with a histological confirmation of infiltrating ductal and lobular breast cancer submitted to radical surgery were eligible. Patients were allowed to have received adjuvant therapy (chemotherapy or hormone therapy) and surgical tumor tissue had to be available for analysis. None of the patients had metastatic disease at time of sample collection (at diagnosis or during surgery), thus had been divided in two subgroups on the base of a minimum follow-up of 10 years.

Fifteen patients with metastatic disease (MET) that had been diagnosed within 10 years of surgery were compared with 16 patients with no evidence of disease (NED) at a minimum follow-up of 10 years for age classes (< 60 years, ≥ 60 years), with an allocation ratio of 1:1. Clinical characteristics of patients are reported in Table 2. All patients were followed up for at least 10 years. No patients had active cardiac disease.

**Table 2:** Patient characteristics

	NED (*n* = 16)	MET (*n* = 15)	Total (*n* = 31)
	No (%)	No (%)	No (%)
**Median age, years (range)**	58 (28–76)	59 (46–81)	59 (28–81)
**Histology**			
Ductal	14 (87.5)	12 (80.0)	23 (74.2)
Lobular	0	3 (20.0)	3 (9.7)
Ductal *in situ*	2 (12.5)	0	2 (6.4)
**Grade**			
1	3 (20)	0	3 (11.5)
2	5 (33.3)	3 (27.3)	8 (30.8)
3	7 (46.7)	8 (72.7)	15 (57.7)
Missing	1	4	5
**Nodal status**			
0	9 (64.3)	3 (33.3)	12 (52.2)
1	5 (35.7)	2 (22.3)	7 (30.4)
2	0	1 (11.1)	1 (4.4)
3	0	3 (33.3)	3 (13.0)
Missing	2	6	8
**Metastatic sites**			
Viscera	–	7 (46.6)	7 (22.6)
Bone	–	4 (26.7)	4 (12.9)
Viscera+bone	–	4 (26.7)	4 (12.9)
NED	–	–	15 (51.6)

NED, no evidence of disease; MET, metastatic disease.

The study was approved by the Ethical Committee of our institute and carried out in accordance with the Declaration of Helsinki. Written informed consent was obtained from all patients to take part in the study.

### Gene expression analysis

Total RNA from MCF10A and HMEi-SNAIL cell line samples collected at the established time-points, and total RNA from primary tissue samples was extracted to perform gene expression analysis of epithelial and mesenchymal genes, EMT-TFs, EMT-related splicing factors and products (Supplementary Table S1). Total RNA from both MCF10A and HMEi-SNAIL were extracted by samples collected at established time points, using RNeasy Mini Kit (Qiagen). Total RNA from primary tissue samples was extracted using the TRIzol reagent (Invitrogen), performed in accordance with manufacturer's instructions. Samples has been treated with DNAsi (Qiagen) to reduce nonspecific detections.

One μg of total RNA was reverse-transcribed into cDNA using the DyNAmo cDNA Syntesis kit (Thermo Scientific). cDNA products were used for RT-qPCR using by different methods. LightCycler^®^ 480 Probes Master Mix (Roche) was used for TaqMan assays design by ProbeFinder software tool (Roche), based on an Universal ProbeLibrary set (UPL), combining a suitable UPL probe with a set of target specific PCR primer pairs for the principal genes analysed (See Supplementary Table S1). Taqman Gene Expression Master Mix (Invitrogen) and SYBR Green with SYBR SELECT Master Mix (Invitrogen) were used respectively to analyse the splicing variant products of FGFR2 (Supplementary Table S1) by specific custom TaqMan Primer/Probe assays, and hMENA genes (Supplementary Table S1). Custom assays for hMNEA splicing variants were performed by PRIMER3 software [52, 53]. qPCR were performed on an ABI7500 Real-Time PCR System (Applied Biosystem). Gene expression levels of each target has been normalized to the expression levels of two reference-gene mRNA (GAPDH and HPRT1), quantifying these two genes for all qPCR methods and chemical reagents used (Supplementary Table S1). Real time analyses were performed as follow: denaturation 95°C for 10 min, denaturation 95°C for 15 sec, annealing, extension, detection at 60°C for 60 sec, 45 cycles. Canonical 2^−(ΔΔCt)^ method has been used to determine the relative expression levels of target genes.

Gene expression-fold changes were reported with respect to basal expression (T0) observed in the two cell lines. All experiments were performed in triplicate.

### Immunohistochemistry

Formalin fixed, paraffin-embedded tissue blocks from 2 NED (NED 1 and NED 2) and 2 MET (MET 1 and MET 2) patients were sectioned 2 μm each and mounted on silane-coated glass slides. Tissues were deparaffinized with xylene followed by rehydration with graded alcohols scaled down to distilled water. One slice per sample was stained using the hematoxylin and eosin method. Two sections per samples were used to detect ESRP1 and RBFOX2 protein expression. These slices, after the rehydration, were submitted to antigen retrieval by incubation with sodium-citrate buffer solution at 98.5°C for 20 min, and then treated with H2O2 for 5 min. Tumor sections were incubated with blocking solution (PBS 1X-BSA 1%) for 30 min, followed by a 20–30 min of cooling period at room temperature, and for one hour at room temperature with primary antibodies diluted in blocking solution. Polyclonal antibody rabbit Anti-human ESRP1diluted 1:100 (Sigma-Aldrich, Human Protein Atlas, HPA023720), 3 μg/ml of mouse monoclonal anti-human RBFOX2, known as Fox2/RBM9 (Abcam). Slide were then incubated with streptavidin-peroxidase conjugate (LSAB+Kit; DAKO Corporation, Carpinteria, CA, USA) for 15 min, washed twice with PBS1X, and stained with diaminobenzidine/hydrogen peroxidase chromogen solution (DAKO+liquid substrate-chromogen solution; DAKO Corporation). Sections were then rinsed in deionised water and counterstained by Mayer's Hematoxylin. All samples were treated with a dehydration step through a growing graded alcohols and xylene for 1 min, and finally mounted by Eukit (Bio-Optica). Sample reactivity was evaluated by light microscopy Axioskop, with optical A-Plan (Zeiss), by two independent observers. Marker positivity was evaluated blindly in a semi-quantitative analysis conferring a grade of expression comprised between 0 and 3+ (0 = negative, 3+ = strongly positive). Imagines in 10X and 20X were captured by digital camera DMC-3a (Polaroid).

### Statistical analysis

Descriptive statistics were reported as proportions and median values. Non-parametric ranking statistics (median test) were used to analyze the relationship between median value of biomarkers and patient status (MET/NED). A volcano plot was used to show statistical significance of different values of gene expression and ratio assays. The horizontal axis represents the difference in median fold change between the two groups (MET and NED) for a single assay, while the vertical axis represents the *p*-value for a *t*-test of differences between samples (on a negative log scale). Assays with a significant differential expression according to the gene-specific *t* test will lie above a horizontal threshold line (dashed line) [54, 55].

Receiver operating characteristic (ROC) curves were used to determine the optimal cut-off values of biomarkers, considered as continuous variables. Sensitivity (the proportion of MET patients correctly identified by a cut-off value < 1.069) and specificity (the proportion of NED patients correctly identified by a cut-off value ≥ 1.069) were calculated. The accuracy of biomarkers was measured using the area under ROC curve (AUC). 95% confidence intervals (95% CI) were calculated for sensitivity, specificity and overall accuracy. All *P* values were based on two-sided testing; to mitigate the issue of multiple testing, a false discovery rate (FDR) of less than 10% was used to determine the relationship between median value of biomarkers and patient status (MET/NED). FDR was controlled using the Benjamini-Hochberg step-up procedure [56]. Statistical analysis was carried out using SAS Statistical Software, version 9.3 (SAS Institute).

## SUPPLEMENTARY MATERIALS TABLES




