# Supplementary material for: Splicing factor ratio as an index of epithelial-mesenchymal transition and tumor aggressiveness in breast cancer
Source: Oncotarget. 2016 Nov 29;8(2):2423–36. doi: 10.18632/oncotarget.13682 (PMC5356812; doi:10.18632/oncotarget.13682)
Supplement: Supplementary file 1 [file oncotarget-08-2423-s001.pdf]

Splicing factor ratio as an index of epithelial-mesenchymal transition and tumor aggressiveness in breast cancer

Supplementary Materials

Supplementary Table S1: Gene expression assays for TaqMan and Sybr green.
See Supplementary_Table_S1
