# Supplementary material for: Splicing factor ratio as an index of epithelial-mesenchymal transition and tumor aggressiveness in breast cancer
Source: Oncotarget. 2016 Nov 29;8(2):2423–36. doi: 10.18632/oncotarget.13682 (PMC5356812; doi:10.18632/oncotarget.13682)
Supplement: Supplementary file 2 [file oncotarget-08-2423-s002.docx]

**Table S1:** Gene expression assays for TaqMan and Sybr green

| **TaqMan assays** | | | | | |
| --- | --- | --- | --- | --- | --- |
| **Gene** | **Entrez Gene Id.** | **Primer fwr** | **Primer rev** | **Probe** | **Ref.** |
| **SNAI1** | **NM_005985.3** | **GCTGCAGGACTCTAATCCAGA** | **ATCTCCGGAGGTGGGATG** | **#11** | **UPL Roche** |
| **SNAI2** | **NM_003068.4** | **TGGTTGCTTCAAGGACACAT** | **GTTGCAGTGAGGGCAAGAA** | **#7** | **UPL Roche** |
| **TWIST1** | **NM_000474.3** | **GGCTCAGCTACGCCTTCTC** | **CCTTCTCTGGAAACAATGACATCT** | **#88** | **UPL Roche** |
| **TWIST2** | **NM_001271893.3** | **CATGTCCGCCTCCCACTA** | **GCATCATTCAGAATCTCCTCCT** | **#10** | **UPL Roche** |
| **ZEB1** | **NM_001128128.2** | **AACTGCTGGGAGGATGACAC** | **TCCTGCTTCATCTGCCTGA** | **#57** | **UPL Roche** |
| **ZEB2** | **NM_001171653.1** | **AAGCCAGGGACAGATCAGC** | **GCCACACTCTGTGCATTTGA** | **#68** | **UPL Roche** |
| **CDH1** | **NM_004360.3** | **CCCGGGACAACGTTTATTAC** | **GCTGGCTCAAGTCAAAGTCC** | **#35** | **UPL Roche** |
| **EPCAM** | **NM_002354.2** | **CCATGTGCTGGTGTGTGAA** | **TGTGTTTTAGTTCAATGATGATCCA** | **#3** | **UPL Roche** |
| **CDH2** | **NM_001792.3** | **CTCCATGTGCCGGATAGC** | **CGATTTCACCAGAAGCCTCTAC** | **#74** | **UPL Roche** |
| **VIM** | **NM_003380.3** | **GACCAGCTAACCAACGACAAA** | **GAAGCATCTCCTCCTGCAAT** | **#39** | **UPL Roche** |
| **FN1** | **NM_001306132.1** | **GCGAGAGTGCCCCTACTACA** | **GTTGGTGAATCGCAGGTCA** | **#52** | **UPL Roche** |
| **ESRP1** | **NM_001122827.1** | **GGCTCGGATGAGAAGGAGTT** | **GCACTTCGTGCAACTGTCC** | **#18** | **UPL Roche** |
| **ESRP2** | **NM_024939.2** | **GCTGTTATCCTCCATCTACTCAAAG** | **GTCCACCACATCAGCCTTG** | **#3** | **UPL Roche** |
| **RBFOX2** | **NM_001082578.1** | **CTCACCCAGCACACAAAATG** | **ACTGCTGGCCGTCTGTCT** | **#39** | **UPL Roche** |
| **HPRT1** | **NM_000194.2** | **TGACCTTGATTTATTTTGCATACC** | **CGAGCAAGACGTTCAGTCCT** | **#73** | **UPL Roche** |
| **GAPDH** | **NM_002046.5** | **CCCCGGTTTCTATAAATTGAGC** | **CACCTTCCCCATGGTGTCT** | **#63** | **UPL Roche** |
| **FGFR2IIIc** | **NM_000141.4** | **CACGGACAAAGAGATTGAGGTTCT** | **CCGCCAAGCACGTATATTCC** | **CCAGCGTCCTCAAAAG** | (45) |
| **FGFR2IIIb** | **M97193.1** | **GGCTCTGTTCAATGTGACCGA** | **GTTGGCCTGCCCTATATAATTGGA** | **TTCCCCAGCATCCGCC** | (45) |
| **HPRT1** | **NM_000194.2** | **ND** | **ND** | **Hs99999909_m1** | **Life Technologies** |
| **GAPDH** | **NM_002046.5** | **ND** | **ND** | **Hs99999905_m1** | **Life Technologies** |
| **Sybr Green assays** | | | | | |
| **GENE** | **Entrez Gene Id.** | **Primer fw** | **Primer Rv** |  |  |
| **ENAH∆v6** | **NM_001008493.1** | **GGGAGAGGCAAGAAAGACAA** | **AAGACAATGCCAGCACTTGATA** | **/** | **Primer3** (52,53) |
| **ENAH11^a^** | **NM_001008493.1** | **GGCAGCAAGTCACCTGTTATCT** | **AATGAATCATAGGACCTGTTGTCA** | **/** | **Primer3** (52,53) |
| **HPRT1** | **NM_000194.2** | **TGAGGATTTGGAAAGGGTGT** | **CTTGAGCACACAGAGGGCTA** | **/** | **Primer3** (52,53) |
| **GAPDH** | **NM_002046.5** | **CCCTTCATTGACCTCAACTACATG** | **TGGGATTTCCATTGATGACAAGC** | **/** | **Primer3** (52,53) |

UPL=Universal Probes Library, Roche; ND= Not defined
